# The consequences of Medicaid expansion under the Affordable Care Act for police arrests

**DOI:** 10.1371/journal.pone.0261512

**Published:** 2022-01-12

**Authors:** Jessica T. Simes, Jaquelyn L. Jahn

**Affiliations:** 1 Department of Sociology, Boston University, Boston, MA, United States of America; 2 Ubuntu Center on Racism, Global Movements, and Population Health Equity, Drexel University Dornsife School of Public Health, Philadelphia, PA, United States of America; George Mason University, UNITED STATES

## Abstract

**Background & methods:**

National protests in the summer of 2020 drew attention to the significant presence of police in marginalized communities. Recent social movements have called for substantial police reforms, including “defunding the police,” a phrase originating from a larger, historical abolition movement advocating that public investments be redirected away from the criminal justice system and into social services and health care. Although research has demonstrated the expansive role of police to respond a broad range of social problems and health emergencies, existing research has yet to fully explore the capacity for health insurance policy to influence rates of arrest in the population. To fill this gap, we examine the potential effect of Medicaid expansion under the Affordable Care Act (ACA) on arrests in 3,035 U.S. counties. We compare county-level arrests using FBI Uniform Crime Reporting (UCR) Program Data before and after Medicaid expansion in 2014–2016, relative to counties in non-expansion states. We use difference-in-differences (DID) models to estimate the change in arrests following Medicaid expansion for overall arrests, and violent, drug, and low-level arrests.

**Results:**

Police arrests significantly declined following the expansion of Medicaid under the ACA. Medicaid expansion produced a 20–32% negative difference in overall arrests rates in the first three years. We observe the largest negative differences for drug arrests: we find a 25–41% negative difference in drug arrests in the three years following Medicaid expansion, compared to non-expansion counties. We observe a 19–29% negative difference in arrests for violence in the three years after Medicaid expansion, and a decrease in low-level arrests between 24–28% in expansion counties compared to non-expansion counties. Our main results for drug arrests are robust to multiple sensitivity analyses, including a state-level model.

**Conclusions:**

Evidence in this paper suggests that expanded Medicaid insurance reduced police arrests, particularly drug-related arrests. Combined with research showing the harmful health consequences of chronic policing in disadvantaged communities, greater insurance coverage creates new avenues for individuals to seek care, receive treatment, and avoid criminalization. As police reform is high on the agenda at the local, state, and federal level, our paper supports the perspective that broad health policy reforms can meaningfully reduce contact with the criminal justice system under historic conditions of mass criminalization.

## Introduction

Recent and widespread police violence protests have renewed debates over the scale of the criminal justice system and the expansive role of police in U.S. communities. These public discussions draw on a longstanding prison abolition tradition that proposes alternative approaches to problems of violence and other criminalized behaviors [1–3]. Well before the hashtag #DefundthePolice went viral, abolitionist activists and scholars called for the redirection of funding from the criminal justice system into a broad set of community resources, particularly non-punitive public services such as health care [4–7]. Abolitionist activists and scholars emphasize that the criminal justice system is currently the default response to problems better addressed through social programs that promote the health and well-being of communities, and that greater public investment in health care and social welfare could reduce the reliance on police in disadvantaged communities.

Police officers are tasked with responding to a wide variety of community concerns including health crises. For example, the reliance on police to enforce social distancing protocols during the COVID-19 pandemic demonstrates the expansive role of police in health emergencies. More broadly, high rates of police contact may reflect the failure of primary prevention policies to meet the basic needs of marginalized populations, particularly in areas related to health, substance use, poverty, and interpersonal conflict [8]. The emergence of what scholars call “mass criminalization” results from two interrelated trends: first, that criminal justice policy fills the gaps left by weak social safety nets in health care, housing, and employment, and second, an unprecedented growth in levels of police contact among the U.S. population [9, 10].

Policing routes millions of people with behavioral health disorders, including substance use problems, into treatment and institutionalization, including jail and prison [11]. Police arrests are a common and pervasive experience in the United States: police make over 10 million arrests per year in the United States, with approximately 1 million for drug-related charges [12]. Following the widespread closure of psychiatric hospitals and the deinstitutionalization of the mental health care system in the late 20th century, state responses to behavioral health problems shifted from one of psychiatric care to control and punishment [11, 13, 14]. Today, 1 in every 5 incarcerated people is locked in jail or prison for a drug charge [15]. A large portion of the population has now been subjected to arrest, which can lead to a cascade of stigmatizing and harmful criminal justice processes. Moreover, limited health insurance coverage suppresses the uptake of community-based behavioral health services and treatment, driving people further into the criminal justice system. In sum, the enormous footprint of the criminal justice system—and the significant precarity and health burdens of the population it affects—present expanded health insurance coverage as a potentially critical measure for reducing levels of police contact in U.S. communities.

In 2010 the Affordable Care Act (ACA) was signed into law by President Obama, with major provisions taking effect in 2014. The goal of the ACA is to broaden health insurance coverage in the United States through a combination of mandates, subsidies, health insurance exchanges, market reforms, and the expansion of Medicaid [16]. The ACA produced historic gains in insurance coverage. The total uninsured population declined from 46.5 million in 2010, to just below 27 million in 2016 [17]. Most of the gains in insurance were among Americans under 65 through the Medicaid expansion provision [17]. Medicaid is the national public health insurance program for people with low income and is the principle source of long-term care coverage for Americans, covering a broad array of health services [18]. Financed mostly by the federal government, the ACA Medicaid expansion provision aims to cover more low-income Americans by expanding the eligibility for Medicaid from previously stricter criteria. However, following a U.S. Supreme Court decision, states could choose to opt into expansion of Medicaid coverage for people up to age 64 with incomes up to 138% of the federal poverty level [16]. At the close of 2021, 12 states had not expanded Medicaid, while 38 states and Washington D.C. had adopted Medicaid expansion [17].

The expansion of health insurance coverage under the ACA generated a large body of research investigating its effects on population health outcomes and health care utilization [19]. Although the majority of substance use disorder treatment is funded through federal block grants from the Substance Abuse and Mental Health Services Administration (SAMHSA)—with around 21% of substance use treatment spending by Medicaid [20], the ACA has also been studied in relation to its effects on opioid treatment receipt and rates of overdose [21]. Recently, scholars have begun to examine the possibility that health policy reform could influence other arenas of social life, including employment, evictions, political participation, crime rates, and migration [22–27]. Two studies have begun to document the ACA’s impacts on criminal justice. One study finds a negative difference in property and violent crime rates after comparing Medicaid expansion and non-expansion states [24]. Another study examines the impacts of Medicaid expansion for the probability of rearrest (i.e. recidivism) in a sample of individuals with a prior conviction in six urban counties, finding mixed results [26]. Relatedly, a large body of health policy research examines how reforms have the potential to simultaneously treat behavioral health conditions and reduce their criminalization [28, 29]. In particular, a host of new practices such as crisis intervention, diversion programs, and comprehensive care programs have sought to reduce the criminalization of people with behavioral health problems [30]. However, no published studies have examined whether health insurance expansion, and specifically Medicaid expansion, affects the level of criminal justice contact faced by the overall U.S. population in a diverse and comprehensive sample of communities. Prior research has also not examined whether expanded health insurance coverage affects particular types of police encounters, such as arrests for drug possession or public disorder.

Taken together, research on criminalization, social inequality, and health policy suggests two key hypotheses that motivate our analysis of the effects of Medicaid expansion on criminal justice contact, and specifically, police arrests. One hypothesis contends that arrests would decline as a result of broader insurance coverage. With the passage of the ACA, expanding insurance coverage to the previously uninsured would lead to greater health care access among those with untreated health care needs in the uninsured population, chiefly among them—mental health and substance use disorder treatment [31–33]. This would in turn reduce the likelihood that police would arrest individuals who may present mental health problems or are involved in drug-related activity or other criminalized behaviors [29, 34–36]. Further, the financial stability gained by providing health insurance to previously uninsured populations could also reduce risk of arrest [27]. By expanding and funding health insurance coverage to millions of previously uninsured people, the ACA may have advanced community health while reducing the reliance on criminal justice institutions that became the dominant response to untreated substance use disorders, mental illness, and poverty in an era of mass incarceration [29, 37]. In this framework, the decision to expand Medicaid insurance coverage should explain a significant reduction in arrests for drug violations in particular, which would suggest that greater access to insurance coverage (and potentially treatment for health problems, for example) removes people from the criminal justice system’s front door.

Conversely, a second hypothesis says that with expansion of Medicaid under the ACA provision, arrests could increase. A substantial body of research supports this hypothesis, suggesting that health policy reforms resulted in either minimal change or even *increased* criminalization of people with behavioral health conditions [38–40]. Increased utilization of treatment and other health care services via expanded insurance coverage could heighten the level of surveillance of participants. This could be due to the spatial concentration of policing near health care institutions that serve low-income populations, or due to the level of data system integration across social service and criminal justice institutions [41, 42]. These factors could increase the chance of surveillance and arrest for those utilizing health care following expanded Medicaid insurance.

## Materials and methods

### Study population

Our aim is to study police arrests comprehensively across U.S. communities. Because arrests are a fundamentally local phenomenon driven by multiple contextual factors, we examine arrest rates in U.S. counties. We remove one outlier county because of its extraordinarily high arrest rate relative to the rest of the sample, providing a final sample size of 3,035 counties (97% of all U.S. counties).

Table 1 reports U.S. states by treatment and control group, and for the treatment group, what year the state implemented Medicaid expansion under the Affordable Care Act. Seven states (Idaho, Maine, Missouri, Nebraska, Oklahoma, Virginia, and Utah) later implemented Medicaid expansion in 2019–2021 but are considered states in the control group because by the end of 2016 (our observation period), they had not yet expanded. Following prior research, we remove the District of Columbia and counties in the following states that expanded Medicaid prior to 2014: Delaware, Massachusetts, New York, and Vermont [43, 44]. We tried models including these states and results are substantively unchanged; we choose to exclude them because early Medicaid expansion policies in these states significantly differ from the ACA.

**Table 1:** Summary of ACA Medicaid expansion adoption as of January 1, 2017.

Treatment Group			Control Group
2014	2015	2016	(No Expansion by January 1, 2017)
Arizona	Alaska	Louisiana	Alabama
Arkansas	Indiana	Montana	Florida
California	Pennsylvania		Georgia
Colorado			Idaho
Connecticut			Kansas
Hawaii			Maine
Illinois			Mississippi
Iowa			Missouri
Kentucky			Nebraska
Maryland			North Carolina
Michigan			Oklahoma
Minnesota			South Carolina
Nevada			South Dakota
New Hampshire			Tennessee
New Jersey			Texas
New Mexico			Utah
North Dakota			Virginia
Ohio			Wisconsin
Oregon			Wyoming
Rhode Island			
Washington			
West Virginia			

*Note*: Idaho, Maine, and Virginia later implemented Medicaid expansion in 2019. Utah expanded in 2020, and Missouri, Nebraska, and Oklahoma expanded in 2021. The District of Columbia and the following states expanded Medicaid prior to 2014 and are not included in the analysis: Delaware, Massachusetts, New York, and Vermont.

This study draws on eight datasets. For arrest data, we use the FBI Uniform Crime Reporting Program Data (UCR) arrest counts from 2011 to 2016 (the most recent available year of data). These data are distinct from the more often used UCR crime counts, which only report Part 1 violent crime (murder and non-negligent manslaughter, rape, robbery, and aggravated assault) and property crime (burglary, larceny-theft, motor vehicle theft, and arson) and include both police-investigated and citizen-reported criminal offenses. Conversely, arrest data are available for 40 offense types and more closely approximate population-level criminalization, as not all reported crime results in an arrest. The UCR counts one arrest for each separate instance in which a person is arrested, cited, or summoned for an offense. Arrest counts are reported voluntarily by law enforcement agencies, aggregated from agencies to the county level, and imputed to address missingness (see Section 2c in S1 File) [45].

For this analysis we explore four arrest categories: all reported arrests, violent arrests (murder, manslaughter, rape, robbery, and aggravated assault), drug arrests (sales and possession), and a combined low-level arrest category that includes both order maintenance (disorderly conduct, prostitution, suspicion, vagrancy, vandalism) and proactive policing (drunkenness, driving under the influence of substances, and possession of stolen property or weapons) [46]. Given our hypothesis about the relationship between behavioral health conditions (especially substance use disorders), insurance coverage, and the risk of arrest, we are particularly interested in how ACA Medicaid expansion affected drug arrests.

We obtained data on county population by race from the U.S. Census Bureau’s Population Estimates Program’s Intercensal County Population Data. We used data on the socioeconomic profile of counties (median age, poverty, and unemployment) from the Robert Wood Johnson Foundation County Health Rankings & Roadmaps and the Census Bureau’s American Community Survey 5-year estimates. We use the National Center for Health Statistics Urban-Rural Classification Scheme to identify county urbanicity. Data on states’ annual education and social welfare expenditures (public assistance, unemployment insurance, housing, and community development) were derived from the Census Bureau Annual Survey of State and Local Government Finances [47]. State-level opioid overdose mortality data were derived from CDC WONDER using the following ICD-10 codes: X40-44, X60-64, X85, and Y10-14. In a sensitivity analysis, we introduce county-level crime data from the UCR into the analysis [45].

Table 2 displays means of variables used in regression analyses. We present means for all counties, and break out data by whether or not states expanded Medicaid. Counties in states that did not expand Medicaid show slightly higher arrest rates overall, and a higher proportion of the population that is Black (12% versus 5%). States that did not expand Medicaid under the ACA tended to spend less per capita on social welfare and education. Rates of opioid-related deaths were about twice as high in non-expansion states.

**Table 2:** Descriptive statistics for variables used in regression analysis of arrests by Medicaid expansion under the Affordable Care Act, 2011–2013.

		All	ACA Expansion	Non-ACA Expansion
All (N = 3,035)	Arrests per 100,000	5321.70	4968.81	5621.48
	Violent arrests per 100,000	148.32	149.4	147.4
	Drug arrests per 100,000	651.78	585.3	708.2
	Low level arrests per 100,000	1285.20	1308.8	1265.2
	Proportion Black	0.09	0.05	0.12
	Median age (years)	40.33	40.79	39.93
	Proportion child poverty	0.24	0.22	0.25
	Proportion unemployed	0.09	0.09	0.09
	Proportion large urban metro	0.13	0.14	0.13
	State welfare spending ($)	1764.77	1973.44	1587.50
	State education spending ($)	993.39	1072.84	925.89
	State opioid deaths per 100,000	8621.73	5106.90	11607.50

*Note*: All variables reported here are measured before treatment.

### Statistical analysis

To evaluate whether Medicaid expansion had an effect on rates of arrest, we use a difference-in-differences (DID) design to estimate the treatment effect of ACA Medicaid expansion on county-level arrests before and after Medicaid expansion in 2014, 2015 and 2016, relative to counties in non-expansion states. We use negative binomial models with an offset of the log total population in counties, which we found using Akaike and Bayesian Information Criteria to have better model fit as compared with Poisson and quasipoisson models. The expected arrest rate *Y*_*it*_, can be written in a negative binomial regression,

$$\log\left(Y_{it}\right)=\beta_{0}+\beta_{1t}+\beta_{2j}+c_{it}^{\prime }\beta_{3}+s_{jt}^{\prime }\beta_{4}+\beta_{5}U_{i}+\delta_{it},$$

where *i* is for counties, *t* is for year, and *j* is for state. $c_{it}^{\prime }$ is a vector of time-varying country-level controls (Black population as a proportion of the total population, poverty rate, unemployment rate, median age). $s_{jt}^{\prime }$ is a vector of time-varying state-level controls for expenditures on welfare and education and the state’s log rate of opioid-related deaths. *U* indicates the level of urbanicity of the county. The DID estimator, *δ*_*it*_, estimates the average change in arrest rate attributable to Medicaid expansion compared to non-expansion states for each year after expansion. Therefore, states that implemented ACA Medicaid expansion in 2016 contribute 1 year of post-expansion observation, 2015 expansion states contribute 2 years of post-expansion observation, and 2014 expansion states contribute 3 years of post-expansion observation. States that did not expand Medicaid by 2016 are set to zero. Finally, *β*_1*t*_ and *β*_2*j*_ are year and state fixed effects, respectively. Thus, our results should be interpreted as the within-state difference in the county-level log rate of arrests per 100,000 residents for each year post expansion compared to pre-Medicaid expansion arrest rates, relative to the log rate difference in counties residing in non-expansion states during those years. Given the presence of heteroskedasticity in our large sample, we estimate confidence intervals for our model estimates using the sandwich estimator [48].

There are several factors that should be considered when examining the relationship between health insurance coverage and arrests. First, arrest patterns vary significantly by local conditions, including concentrated disadvantage and racial segregation, and the spatial organization of criminalized behavior [49, 50]. Certain types of arrest—such as arrests for drug or low-level disorder charges—are associated with populations beset by untreated substance use problems, mental illness, and economic deprivation [51]. State-level factors, such as the level of social welfare spending, could also offset the role that criminal justice institutions play in the administration of health care [37]. We hypothesize that demographic, socio-economic, and health factors, as well as social welfare spending, could contribute to observed differences in police arrests and states’ decision to expand Medicaid. For example, counties with high levels of poverty and unemployment, and where a greater share of the population is young and Black, may have higher arrest rates [52–54] and reside in states less inclined to opt-in to Medicaid expansion [55]. Additionally, states with lower per capita expenditures on social welfare may have higher arrest rates and be less inclined to expand Medicaid [24].

Beyond theories of criminalization, arrest rates could also have been affected by the opioid epidemic that began in the early 2000s. Nationally, opioid deaths quadrupled between 1999 and 2015, precisely during the period of Medicaid expansion under the Affordable Care Act [21, 56]. Arrests may have increased due to greater drug enforcement or decreased as states sought to expand treatment services due to the epidemic. Moreover, a state’s decision to expand Medicaid could be influenced by its approach to addressing the opioid crisis.

To account for these potential confounders, we control county-level median age, unemployment rate, poverty rate, and Black resident population as a percentage of the total population. We use state-level measures of opioid overdose mortality and investments in social welfare and education (in 2017 dollars) [21, 24]. We tried models that estimate state political context, but with the inclusion of state fixed effects, we determined these highly collinear and did not include measures of state political context in our models. In addition to county and state-level controls and state and year fixed effects, we include an additional control for the county’s level of urbanicity (large metropolitan areas, small/medium metropolitan areas, and rural areas). We explore controlling for county-level crime rates in a sensitivity analysis, but due to concerns about post-treatment bias, our main DID models do not adjust for crime rates.

### Sensitivity analyses

There are two core assumptions of the DID approach. First, we must show that trends in Medicaid expansion county outcomes (treatment group) during this time period would be similar to non-expansion counties (control group) had they not expanded Medicaid. The second major identifying assumption of DID is that there are no confounding conditions affecting arrests at the same time as Medicaid expansion. We conduct a set of sensitivity analyses to test the robustness of our results to these two main assumptions. In Section 1 of S1 File, we conduct placebo tests to evaluate whether the changes in arrests between expansion and non-expansion states after ACA Medicaid expansion show a divergence in rates of arrest across treated and control counties pre-dating the ACA. To do so, we shift the “expansion” year backwards in time for each year between 2000 and 2012 and compare changes in arrests between expansion and non-expansion states.

We then compare our main models to an analysis using propensity score matching, where counties are matched based on the same pre-treatment covariates used in the DID analysis (Section 2a in S1 File). Matching aims to address bias associated with selection into treatment but limits the precision and generalizability of our estimates because counties are discarded through the matching procedure. Next, we assess whether changes in arrests after Medicaid expansion are explained by changes in criminalized behaviors. Arrest rates could have changed due to shifts in criminalized behavior in the population, shifts in policing, or a combination of both. We adjust our DID models for annual county-level crime rate (Section 2b in S1 File), but our main models do not adjust for crime to avoid post-treatment bias. Finally, we evaluated whether our DID findings were biased due to missing data or level of aggregation. We provide a discussion of data coverage and missingness in the UCR arrest data in Section 2c of S1 File. Following prior research, we replicate our analysis with data aggregated to states (Section 2c in S1 File) [24].

## Results

**Fig 1:**
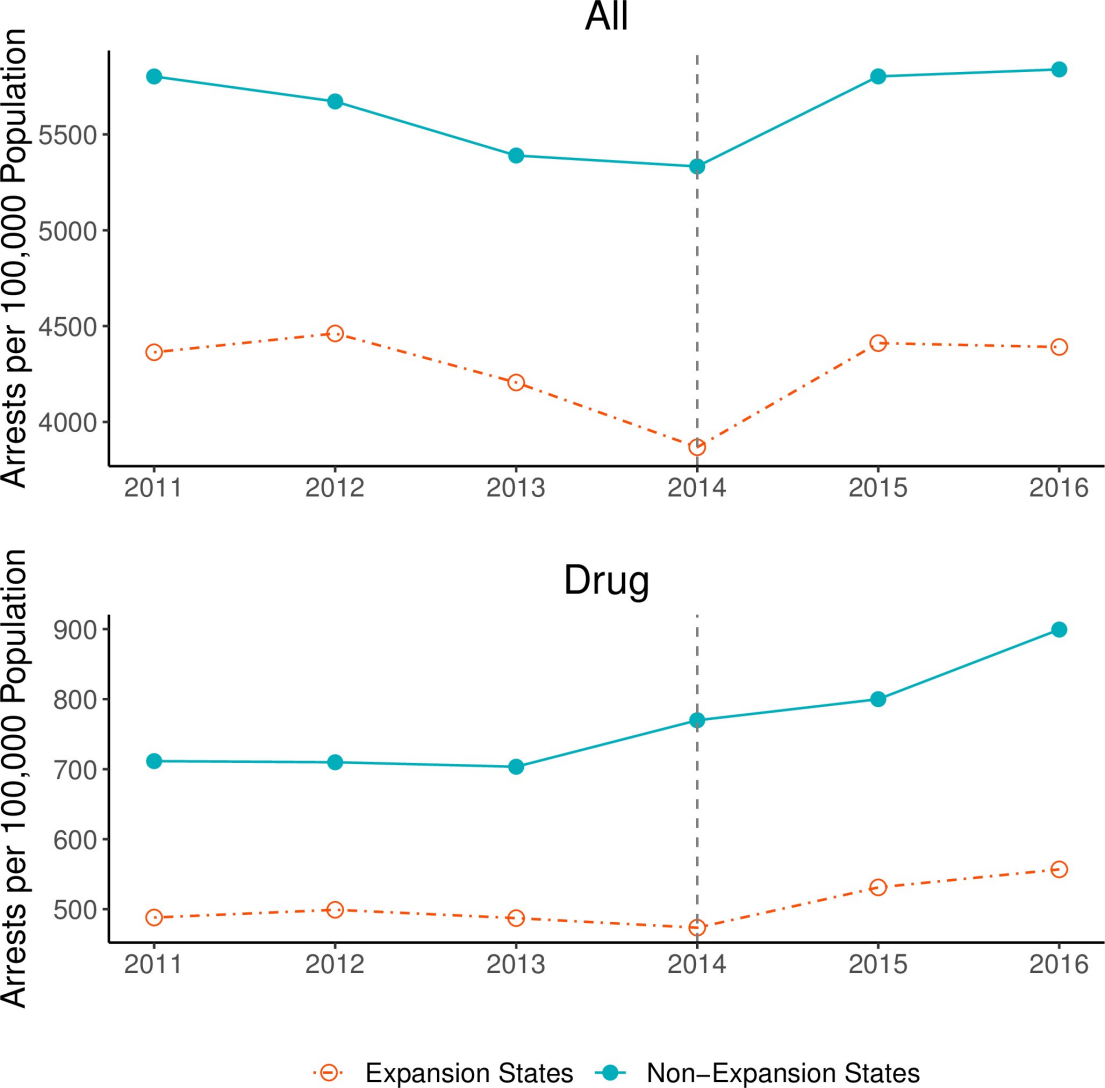
Rates of all arrests and drug arrests for analysis years, 2011–2016. We plot the total and drug arrest rates per 100,000 residents in counties, unadjusted by covariates and exclude areas that expanded Medicaid before or after 2014. A vertical line indicates the first year ACA Medicaid expansion took effect (2014).

Baseline levels of all and drug arrests were higher in non-expansion states, but the trends in arrest rates before expansion (years 2011–2013) were similar (Fig 1). After expansion, arrests increased in both expansion and non-expansion states. We observe an increase for overall arrest rates and drug arrests over this time period. Indeed, post-2014 rates of drug arrest in non-expansion states increased, and although drug arrests also increased in expansion states, the rate of increase appears to be less steep than that of non-expansion states.

**Fig 2:**
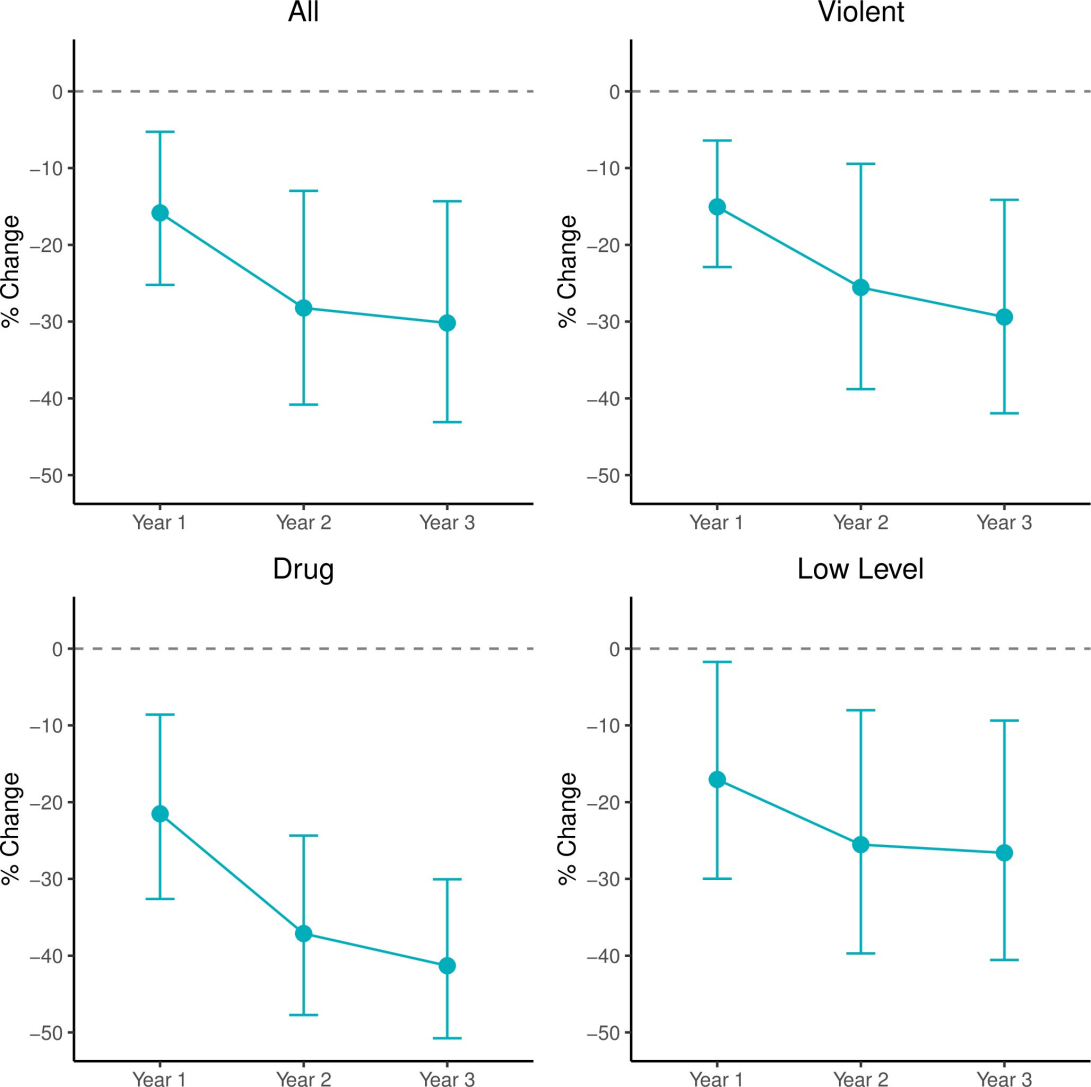
Estimated percentage change and 95% confidence intervals in arrest rates each year after Medicaid expansion, compared to non-expansion counties. Estimates from difference-in-differences models comparing changes in arrest rates each year after Medicaid expansion, compared to non-expansion counties. These models include county and state-level covariates as well as state and year fixed effects.

Fig 2 displays the difference-in-differences (DID) negative binomial regression results for each of the four arrest categories: all arrests, drug arrests, violent arrests, and low-level arrests. In Fig 2, we plot the estimated percentage change in arrests for each year after Medicaid expansion and 95% confidence intervals compared to non-expansion counties. (Results for covariates are reported in S1 Table in S1 File.) Overall, the results show a striking effect of ACA Medicaid expansion on arrests. All coefficient estimates are negative and statistically significant. These results provide support for our hypothesis that ACA Medicaid expansion is associated with a negative difference in arrests.

In models predicting all arrests (Fig 2), we estimate a 20–32% negative difference in the rate of arrest in counties in expansion states compared to counties in non-expansion states (p < .01). This effect is persistent in all three years of expansion, controlling for county-level urbanicity level, racial composition, unemployment, state-level opioid-related mortality rates and welfare spending, and all time-invariant state-level confounders.

When we disaggregate by arrest type, we find significant negative differences in arrests for violent, drug, and low-level arrests. We observe a significant 19–29% negative difference in arrests for violent arrests in all three years of Medicaid expansion (p < .01). We observe a decrease in low-level arrests between 24–28% in expansion states compared to non-expansion states (p < .01).

Finally, we estimate comparatively large negative differences in drug arrests following the implementation of the Medicaid expansion provision of the Affordable Care Act. In all three years after the Medicaid expansion provision took effect, county-level drug-related arrests in expansion counties saw a negative difference of between 25 and 41% relative to counties in non-expansion states (p < .01).

### Sensitivity analysis results

Our first set of sensitivity analyses used placebo tests (S1 Fig in S1 File) to assess whether pre-ACA trends in arrests differed across expansion and non-expansion counties. All results from the placebo tests are null, indicating states that eventually expanded Medicaid showed no relative difference in arrests in the years immediately prior to the implementation of the ACA.

Our second sensitivity analysis examined common support in the sample of expansion and non-expansion counties (S2 Fig in S1 File), and for relevant time-varying confounding that could affect arrests or influence the decision to expand Medicaid under the ACA. Results using DID Propensity Score Matching for all arrest types were similar to our main results (S3 Fig in S1 File). Results were substantively unchanged after adjusting for county-level crime rates, suggesting that arrest rates declined net of changes to reported crime in counties (S3 Fig in S1 File).

We also evaluated patterns of missingness in the available UCR data. Results suggest counties with higher levels of non-reporting had higher imputed arrests rates compared to counties with nearly complete reporting, but were not more likely to expand Medicaid in 2014 (S2 Table in S1 File). When we repeated our main DID models excluding 448 counties (1,915 county-years) for which data were entirely imputed, we found relative decreases for drug arrests consistent with our main findings, but results for other arrest types were null (S3 Fig in S1 File).

Lastly, as an additional test for the potential bias from county-level estimates, we replicated our model in a state-level framework and find significant negative differences in drug arrests, consistent with our main findings, but associations for other types of arrests were null (S3 Fig in S1 File).

## Discussion

In a series of difference-in-differences (DID) negative binomial regression models, ours is the first study to explore the effect of Affordable Care Act Medicaid expansion on arrests in a near-complete sample of U.S. counties. Previous research evaluating Medicaid expansion on criminal justice outcomes has focused on crime or recidivism in more limited samples [24, 26]. Expanding Medicaid insurance coverage could have led to greater health care access and thus reduced criminal justice contact; conversely ACA Medicaid expansion could have increased the surveillance and arrest of those with untreated health care needs in the uninsured population. We find evidence to support the hypothesis that expanding insurance coverage reduces the level of criminalization experienced broadly within communities. We observe this effect in a diverse set of police encounters, including violent and drug arrests.

Following sensitivity analyses, we find the strongest evidence for the effect of Medicaid expansion for drug arrests. Models show significant negative differences in county-level drug arrests in all three years after expansion in our main results and across all sensitivity analyses. These results suggest that Medicaid expansion may have been particularly important for providing insurance coverage for individuals with substance use problems and reducing their contact with the criminal justice system. The ACA took effect during a period when drug overdose deaths sharply increased [56]. Prior research found that Medicaid expansion was associated with fewer opioid overdose deaths at the county level, particularly for deaths involving heroin and synthetic opioids other than methadone [21]. Medicaid expansion also increased insurance coverage among people with heroin use disorders, but may not have increased uptake of treatment in this population [57]. While there is growing evidence suggesting which types of substance use disorder treatment reduce arrests [35, 58, 59], future research should assess whether our finding of the negative difference in drug arrests following Medicaid expansion was due to increased access to substance use treatment, or whether our findings are explained by other mechanisms such as improved financial wellbeing following insurance coverage [27]. Although such analyses were not possible with the publicly-available data used in this study, our results suggest changes to health insurance policy reduced the criminalization of substance use problems.

Our findings highlight the potential for health insurance policy to affect population-level criminal justice contact. In counties where there may have been an over-reliance on policing to respond to individuals with health problems, insurance coverage creates new avenues for individuals to seek care, receive treatment, and avoid arrest. Our findings contribute to an understanding of how social policy can prevent cumulative disadvantage following from health problems to arrest and improve health for marginalized and chronically policed populations.

## Conclusions

Our results support the conclusion that health policy could meaningfully reduce the footprint of the criminal justice system. However, we caution that relying on social and health policy alone to decrease police arrests in communities could prove precarious. In 2019, federal courts struck down the insurance mandate of the ACA, and the U.S. Supreme Court has received additional challenges to the Affordable Care Act. Eleven years after its passage, the ACA faces political and legal challenges despite a recent Kaiser poll finding that 55% of the public supports the policy [17].

Although evidence presented in this paper suggests ACA Medicaid expansion is associated with a negative relative difference in arrests, overall arrest rates nevertheless *increased* on average in the period following ACA expansion, even in states that expanded Medicaid under the ACA (see Fig 1). Thus, we believe a combination of health policy, social policy, and criminal justice policy reform will together reduce the over-reliance on policing in communities. Policing and jails in the United States play a significant role in managing health problems among people living in poverty [13, 60]. Moreover, contact with the criminal justice system has been widely shown to reproduce social inequality, exacerbate physical and psychiatric health conditions, and may be especially burdensome in an infectious disease pandemic [37, 61–63]. The current study has significant implications for the possible collateral consequences of the COVID-19 pandemic, in which reports have shown that the reliance on the police to enforce public health ordinances has created another avenue in which the pandemic may have impacted disadvantaged communities [64, 65].

We find evidence that Medicaid expansion impacted criminal justice practice by significantly reducing arrests, particularly for drug selling and possession. With this evidence we believe that broad access to insurance coverage can reduce police contact. However, for these effects to be fully realized and long-lasting, we believe that a broad set of social welfare and criminal justice policy reforms will be necessary to meaningfully reduce criminalization in the United States.

## Supporting information

S1 File (DOCX)

## Abbreviations

ACA: Affordable Care Act
FBI: Federal Bureau of Investigation
UCR: Uniform Crime Reports
DID: Difference-in-Differences
