# Supplementary material for: The consequences of Medicaid expansion under the Affordable Care Act for police arrests
Source: PLoS One. 2022 Jan 12;17(1):e0261512. doi: 10.1371/journal.pone.0261512 (PMC8754343; doi:10.1371/journal.pone.0261512)
Supplement: S1 File — (DOCX) [file pone.0261512.s001.docx]

**The Consequences of Medicaid Expansion Under the Affordable Care Act for Police Arrests**

Jessica T. Simes^1^*; Jaquelyn L. Jahn^2^

^1^ Department of Sociology, Boston University, Boston, MA, USA; ^2^ CUNY Graduate Center, New York, NY, USA.

* Corresponding author

Email: [simes@bu.edu](mailto:simes@bu.edu)

Supporting Information

**Section 1: Placebo Tests to Evaluate Parallel Trends in Pre-Expansion Years**

S1 Fig displays point estimates and 95% confidence intervals from a set of earlier-in-time placebo models that set the year of treatment to every year between 2000 and 2012 (before ACA was implemented in 2014). In doing so, we follow previous literature evaluating the ACA [1]. We compare whether expansion and non-expansion states had differing trends in arrests (overall and stratified by type) in the years prior to expansion, or whether the post-expansion differences in trends reflect a broader secular divergence in arrest patterns that pre-date the ACA. We find no statistically significant changes in arrests between expansion and non-expansion states in the years prior to the passage of the ACA, indicating that the changes in arrests post-ACA reflect a new divergence between expansion and non-expansion states. Note that in S1 Fig, point estimates appear to be exactly zero given large confidence intervals but are in fact all different values slightly less than zero.

**Section 2: Comparison of Estimation Strategies**

There are two core assumptions of the DID estimation strategy: parallel trends and the absence of time-varying confounding. In addition, our results may be biased due to missing data or the choice of geographic unit of analysis (i.e. counties). To test the robustness of our results to these potential threats to causal inference, we compare the following estimation strategies with the DID analysis reported in the main paper:

1. DID Propensity Score Matching (PSM) analysis (described in 2a below)
2. DID model in the main paper additionally adjusting for the county’s crime rate (described in 2b below)
3. DID analysis that adjusts for bias associated with missing arrest data or data aggregation (described in 2c below)

**Section 2a: Difference-in-Differences Propensity Score Matching (DID-PSM)**

S2 Fig displays histograms of the estimated propensity scores by treatment status: ACA expansion and non-expansion. While we observe common support, we also observe a strong pattern indicating treatment status varies with the probability of treatment (i.e., treated counties reside in a state that would have likely expanded Medicaid under the provision of the ACA).

To test the parallel trends assumption of the DID, we conducted propensity score matching that matched counties on pre-treatment covariates. We used the MatchIt package in R to estimate these results [2]. The matching algorithm employs nearest neighbor matching without replacement using the same pre-treatment covariates from the DID models presented in the paper. There is a bias/variance trade off in deciding to match with or without replacement. We explored matching with and without replacement and find that results are similar using either method.

S3 Fig displays results from DID-PSM analyses. The results show significant effects of the ACA for all arrest types in the matched sample—consistent with the DID results reported in the main analysis (Fig 2). We find a negative difference of 17–31% in all arrests in expansion state counties relative to non-expansion states in the matched sample. The drug arrest model shows a similar striking decline in arrests in all three years of expansion (IRR in Year 1: .77, CI: .66, .90).

**Section 2b: DID Models Adjusting for County-Level Crime Rate**

Prior research has examined the impacts of ACA on crime and recidivism [3,4]. One possible explanation for declining arrests is that reported crime declined at the same time that ACA Medicaid expanded, a source of time-varying confounding. S3 Fig displays estimates from DID models that adjust for county-level crime rates (total crimes as reported in the UCR). The results suggest that adjustment for crime rate does not substantively change the estimates reported in our main models. Due to the potential for post-treatment bias in models that predict arrests with crime rate, our main results (Fig 2) do not adjust for crime.

**Section 2c: Missing Data and Aggregation in the UCR Arrest Data**

There are reported issues with missingness in arrest data efforts in the United States [5]. County-level arrest data from the Uniform Crime Reports (UCR) were originally collected by the Federal Bureau of Investigation from monthly reports submitted voluntarily by law enforcement agencies. To address this missing data problem, we used agency-level arrest counts that were aggregated to the county level by Jacob Kaplan [6]. For agencies that did not report any data or reported less than 12 months of data, Kaplan applied a missing data strategy that was designed by the National Archive of Criminal Justice Data. We examine the distribution of key study variables across levels of the degree of imputation within a county. We find that counties with more imputed data had higher rates of arrest, but were not more likely to expand Medicaid in 2014. Counties with more imputed data had higher proportions of Black residents, higher levels of child poverty and unemployment, and resided in states with lower levels of spending on welfare and education, and lower rates of opioid mortality (S2 Table).

S4 Fig displays a histogram of the coverage indicator, which is the proportion of data within a county that is imputed for a given year, with higher values indicative of more coverage (and less imputed data) and lower values indicative of less coverage (and more imputed data). There were 1,915 county-years (448 counties) with a coverage indicator of 0 indicating completely imputed data.

We replicated the DID models from our main analysis excluding counties with 100% imputed data. S3 Fig displays the estimated percentage change and 95% confidence intervals comparing arrests by type for expansion versus non-expansion states for each year post expansion, excluding counties that had a coverage indicator of 0. The results suggest that the negative decreases in drug arrests in the main analysis are consistent in this subset of counties. However, our findings for total, violent and low-level arrests are null in this sample.

S3 Fig also includes a state-level analysis to assess whether our main county-level findings hold at this alternative geographic scale, following prior research [3]. We find at the state-level that drug arrests decreased in states that expanded relative to states that did not expand Medicaid. Rates of arrest vary substantially within states at the county level due to localized patterns of policing and multiple contextual factors. Although the ACA’s Medicaid expansion is a state-level policy, our state-level findings suggest that the policy likely had localized effects that can be masked by state-level averages.

**References**

1. J G Slusky D. Significant Placebo Results in Difference-in-Differences Analysis: The Case of the ACA’s Parental Mandate. Eastern Econ J. 2017 Sep 1;43(4):580–603.

2. Stuart EA, Huskamp HA, Duckworth K, Simmons J, Song Z, Chernew M, et al. Using propensity scores in difference-in-differences models to estimate the effects of a policy change. Health Serv Outcomes Res Methodol. 2014 Dec 1;14(4):166–82.

3. He Q, Barkowski S. The effect of health insurance on crime: Evidence from the Affordable Care Act Medicaid expansion. Health Economics. 2020;29(3):261–77.

4. Fry CE, McGuire TG, Frank RG. Medicaid Expansion’s Spillover to the Criminal Justice System: Evidence from Six Urban Counties. RSF. 2020 Jul;6(2):244–63.

5. Maltz MD, Targonski J. A Note on the Use of County-Level UCR Data. Journal of Quantitative Criminology. 2002 Sep 1;18(3):297–318.

6. Kaplan J. Jacob Kaplan’s Concatenated Files: Uniform Crime Reporting Program Data: Offenses Known and Clearances by Arrest, 1960-2019 [Internet]. Open Inter-university Consortium for Political and Social Research. Inter-university Consortium for Political and Social Research (ICPSR); 2021 [cited 2021 Jul 2]. Available from: https://www.openicpsr.org/openicpsr/project/100707/version/V16/view;jsessionid=52480A9CA85D0DE54EC5535A568A6845

**S1 Table. Regression coefficients for covariates in difference-in-differences negative binomial regression models of percentage change in arrest rates following Medicaid expansion.**

|  |  | All | Violent | Drug | Low Level |
| --- | --- | --- | --- | --- | --- |
| *County-level estimates* | |  |  |  |  |
|  | Prop. Black | .702*** | 1.332*** | .610*** | .545*** |
|  |  | (.532, .873) | (1.185, 1.478) | (.442, .779) | (.367, .724) |
|  | Median age | .0005 | .002 | .001 | -.003 |
|  |  | (-.004, .005) | (-.002, .005) | (-.003, .005) | (-.008, .002) |
|  | Prop. child poverty | 1.219*** | 1.998*** | .920*** | 1.019*** |
|  |  | (.825, 1.613) | (1.716,2.280) | (.541, 1.299) | (.646, 1.392) |
|  | Prop. unemployed | -1.732*** | -1.031* | -2.668*** | -1.505* |
|  |  | (.532, .873) | (-1.901,-.162) | (-3.590, -1.745) | (-2.687, -.324) |
|  | Rural metro | -.164*** | -.152*** | -.241*** | -.148*** |
|  |  | (-.242, -.085) | (-.207,-.098) | (-.305, -.178) | (-.212, -.084) |
|  | Small metro | .103 | .023 | .042 | .099 |
|  |  | (-.028, .235) | (-.101, .146) | (-.085, .170) | (-.045, .243) |
| *State-level estimates* | |  |  |  |  |
|  | Welfare spending | .001** | .0007** | .001*** | .001* |
|  |  | (<0.001,0.001) | (<0.001,0.001) | (<0.001,0.001) | (<0.001,0.001) |
|  | Education spending | .0004* | .0005* | .0003 | .0004 |
|  |  | ((<0.001,0.001) | (<0.001,0.001) | ((<0.001,0.001) | (<0.001,0.001) |
|  | Log opioid death rate | .157*** | .059*** | .200*** | .187*** |
|  |  | (.109, .205) | (.024,.093) | (.161, .238) | (.146, .228) |
|  |  |  |  |  |  |
| *N* Counties | | 3,035 | 3,035 | 3,035 | 3,035 |

* p<.05 ** p<.01 *** p<.001

*Note:* Robust 95% confidence intervals in parentheses. Models include year and state fixed effects not shown.

**S2 Table. County and state measures across the coverage indicator (CI) in the UCR arrest data, 2011-2016.**

|  | CI ≤ 25 | CI > 25 & ≤ 75 | CI > 75 | P-value |
| --- | --- | --- | --- | --- |
| Dependent variable  Arrests per 100,000 | 8183 | 6265 | 4390 | 0.001 |
| Treatment status  Non-expansion (%)  Expanded in 2014 (%)  Expanded in 2015 (%)  Expanded in 2016 (%) | 54.9  36.7  5.9  2.6 | 49.1  36.6  6.8  7.5 | 54.9  35.9  5.7  3.5 | < 0.001  0.68  0.08  < 0.001 |
| County-level measures  Total population  Prop. Black  Median age (years)  Prop. child poverty  Prop. unemployed  Prop. large urban metro | 93,459  0.12  41.7  0.25  0.09  0.12 | 40,954  0.09  42.1  0.24  0.07  0.05 | 106,831  0.09  40.1  0.24  0.08  0.15 | < 0.001  < 0.001  < 0.001  < 0.001  < 0.001  < 0.001 |
| State-level measures  Welfare spending ($)  Education spending ($)  Opioid deaths per 100,000 | 1718  824  15,928 | 1758  1058  17,370 | 1786  1014  6226 | < 0.001  < 0.001  < 0.001 |

*Note*: CI=Coverage indicator, which is the percentage of data within a county that is reported for a given year, with higher values indicative of more coverage (and less imputed data), and lower values indicative of less coverage (and more imputed data). P-values are from a one-way ANOVA to compare means of continuous variables across groups and a chi-square test for categorical variables.

**S1 Fig. Placebo tests to evaluate parallel trends in pre-expansion years, 2001–2011.**

*Note*: Point estimates appear to be exactly zero given the wide confidence intervals but are all different values slightly less than zero.

**S2 Fig. Estimated propensity score by treatment status: ACA expansion and non-expansion, 2014-2016.**


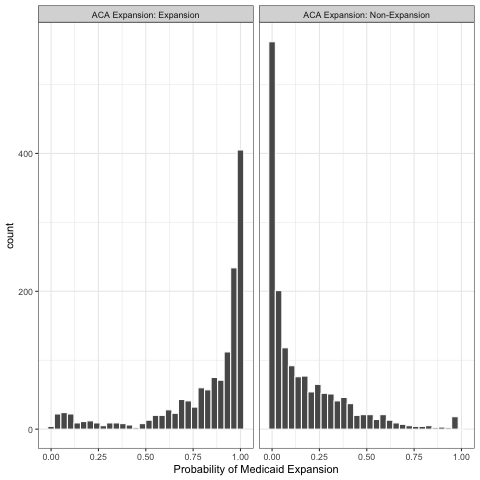


**S3 Fig. Estimated percentage change and 95% confidence intervals comparing arrests by type for expansion and non-expansion areas for each year post estimation across different modeling strategies.**

**Year 1**

**
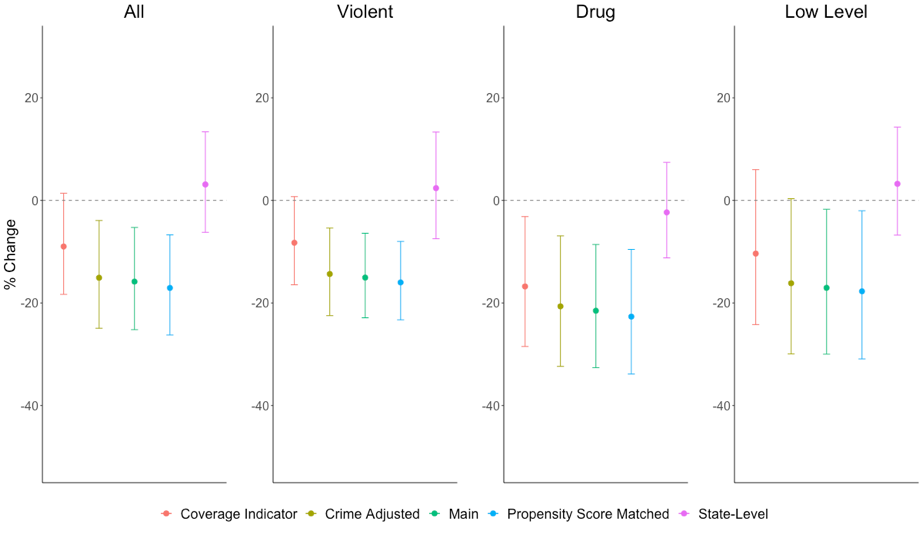
**

**Year 2**

**
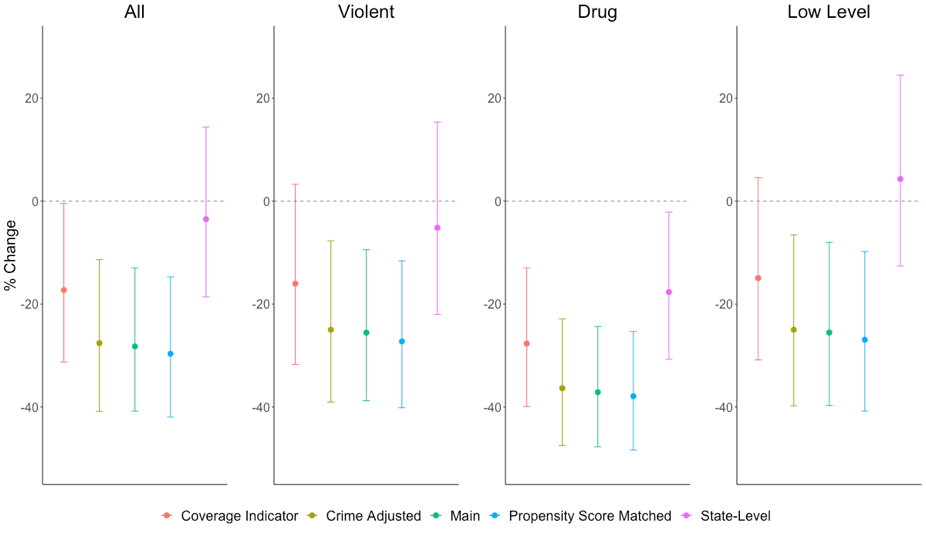
**

**Year 3**

**
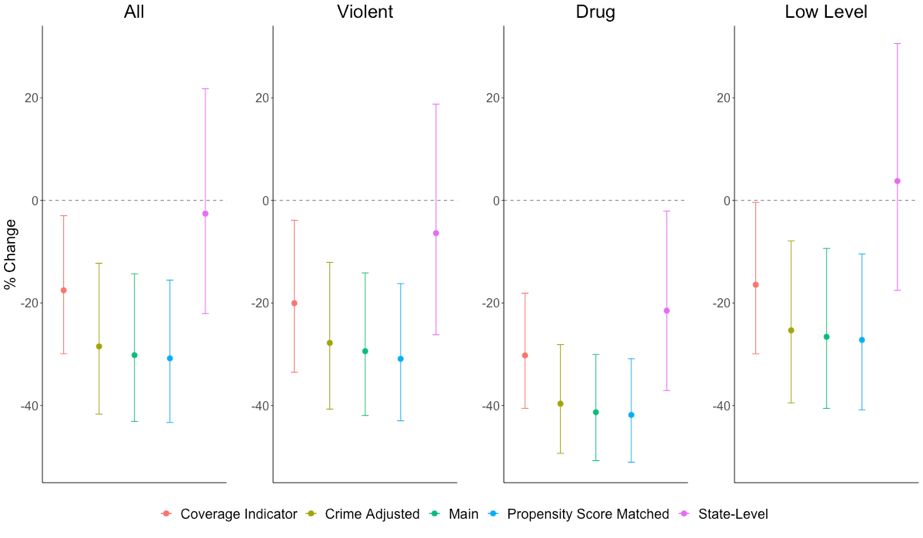
**

**S4 Fig. Histogram of the coverage indicator for non-imputed UCR arrest data, 2011-2016.**

*Note*: The coverage indicator in UCR arrest data indicates the proportion of data within a county that is imputed for a given year, with higher values indicative of more coverage (and less imputed data) and lower values indicative of less coverage (and more imputed data).
